# PROSPECT: 4- and 6-year follow-up of a randomised trial of surgery for vaginal prolapse

**DOI:** 10.1007/s00192-022-05308-0

**Published:** 2022-08-26

**Authors:** Fiona M. Reid, Lorna Aucott, Cathryn M. A. Glazener, Andrew Elders, Christine Hemming, Kevin G. Cooper, Robert M. Freeman, Anthony R. B. Smith, Suzanne Hagen, Mary Kilonzo, Dwayne Boyers, Graeme MacLennan, John Norrie, Suzanne Breeman, [for the PROSPECT study group]

**Affiliations:** 1 Warrell Unit, St Mary’s Hospital, Manchester University Hospitals NHS Foundation Trust, Manchester Academic Health Science Centre, Oxford Road Campus, Manchester, M13 0JH UK; 2 Health Services Research Unit, University of Aberdeen, Aberdeen, UK; 3 NMAHP Research Unit, Glasgow Caledonian University, Glasgow, UK; 4 Department of Obstetrics and Gynaecology, Aberdeen Royal Infirmary, Aberdeen, UK; 5 Department of Obstetrics and Gynaecology, Plymouth Hospitals NHS Trust, Plymouth, UK; 6 Health Economics Research Unit, University of Aberdeen, Aberdeen, UK; 7 Edinburgh Clinical Trials Unit, Usher institute, University of Edinburgh, Edinburgh, UK

**Keywords:** Polypropylene mesh, Prolapse, Randomised trial, Xenograft

## Abstract

**Introduction and hypothesis:**

Our aim was to compare the mid-term results of native tissue, biological xenograft and polypropylene mesh surgery for women with vaginal wall prolapse.

**Methods:**

A total of 1348 women undergoing primary transvaginal repair of an anterior and/or posterior prolapse were recruited between January 2010 and August 2013 from 35 UK centres. They were randomised by remote allocation to native tissue surgery, biological xenograft or polypropylene mesh. We performed both 4- and 6-year follow-up using validated patient-reported outcome measures.

**Results:**

At 4 and 6 years post-operation, there was no clinically important difference in Pelvic Organ Prolapse Symptom Score for any of the treatments. Using a strict composite outcome to assess functional cure at 6 years, we found no difference in cure among the three types of surgery. Half the women were cured at 6 years but only 10.3 to 12% of women had undergone further surgery for prolapse. However, 8.4% of women in the mesh group had undergone further surgery for mesh complications. There was no difference in the incidence of chronic pain or dyspareunia between groups.

**Conclusions:**

At the mid-term outcome of 6 years, there is no benefit from augmenting primary prolapse repairs with polypropylene mesh inlays or biological xenografts. There was no evidence that polypropylene mesh inlays caused greater pain or dyspareunia than native tissue repairs.

**Supplementary Information:**

The online version contains supplementary material available at 10.1007/s00192-022-05308-0.

## Introduction

There has been concern about the long-term effectiveness of prolapse surgery and its complications, the limited available evidence suggesting failure rates of up to 50% [1, 2] and re-operation rates up to 30%, albeit this included continence surgery too. The mean time interval to the second operation was 12 years [3]. Although prolapse can affect young women, it is a disease associated with ageing. The number of adults > 65 years doubled between 1980 and 2020 [4] and the numbers globally living to become centenarians is predicted to rise by ten-fold by 2050 [5]. Hence, considering the failure rates of surgery for prolapse and the increasing ageing population, it is important to find a surgical solution with longevity and with a low complications rate.

To improve outcomes following prolapse surgery, polypropylene mesh and biological grafts have been used. However, evidence to justify these new approaches was absent, unreliable or conflicting. In 2017 the findings from PROSPECT, a randomised controlled trial (RCT) that compared polypropylene mesh inlays, biological xenografts and native tissue surgery for vaginal prolapse in 1348 women undergoing primary prolapse surgery, showed that augmentation of a vaginal repair with polypropylene mesh inlays or biological xenograft material did not improve outcomes at 2 years [6]. In a secondary analysis of the complete cohort of women in PROSPECT (*n* = 2632) we found low surgical morbidity and low rates of severe complications following any prolapse surgery [7]. Nevertheless, we acknowledged that the findings in the longer term may still overturn these conclusions. Hence, mid- and long-term follow-up is needed.

Also, over the past decade anecdotal evidence has emerged suggesting that some women who received polypropylene mesh to augment their prolapse surgery experienced severe and debilitating adverse effects [8]. Some of these complications may have delayed onset. However, there is little evidence about the relative risks of these conditions occurring following mesh compared to native tissue or biological xenograft surgery.

This article reports on the mid-term follow-up, at 4 and 6 years, of women randomised within PROSPECT undergoing primary prolapse surgery.

## Methods

### Participants

Women undergoing primary transvaginal repair of an anterior and/or posterior prolapse were enrolled into the trial between January 2010 and August 2013 from 35 UK centres. All women provided written informed consent to participant. Baseline data, operative details, outcome data to 2-years following surgery, types of mesh and xenograft used and full methodology of the trial have been reported previously [6, 9].

Women were recruited and randomised within three strata: Stratum A included women randomised among all three treatment options: native tissue repair, polypropylene mesh inlay and biological xenograft (in a 1:1:1 ratio); Stratum B included women randomised between native tissue repair and polypropylene mesh inlay (in a 1:1 ratio); Stratum C included women randomised between native tissue repair and biological xenograft (in a 1:1 ratio).

### Outcomes

In this longer-term follow-up period, women were followed up at 4 and 6 years after surgery by postal questionnaire. Those women who did not have surgery in the initial 2-year period were followed by postal questionnaire at 4 and 6 years post-randomisation.

In keeping with IUGA/ICS recommendation [10], the primary outcome was participant-reported prolapse symptoms at 6 years after surgery using the Pelvic Organ Prolapse Symptom Score (POP-SS) [11]. Secondary outcomes included prolapse-specific quality-of-life measured using a visual analogue scale (VAS), generic quality of life based on the EQ-5D-3L [12] and an assessment of overall global improvement in symptoms (PGI-I) [13]. Bladder, bowel and sexual functions were measured using validated or adapted International Consultation on Incontinence Questionnaires (ICIQ) [14].

Further surgical treatment and conservative treatment for prolapse recurrence, need for mesh removal surgery and other related hospital readmission were reported by women in their postal questionnaires.

### Statistical analysis

The main analysis was conducted on an intention-to-treat basis (whereby women with observed outcome data remained in their allocated group for analysis). Two comparisons were made: native tissue repair versus polypropylene mesh inlay (Trial 1, data from women in Strata A and B) and native tissue repair versus biological xenograft (Trial 2, from women in Strata A and C). As the analyses were carried out for Trial 1 and Trial 2 separately, data from some women in the native repair arm from Stratum A were included in both trials. Study analyses were conducted according to a prespecified statistical analysis plan, using SAS version 16.1 (SAS Institute Inc., Cary, NC, USA).

All outcome measures were presented as summaries using descriptive statistics (mean and standard deviation for continuous measures and proportion for ordinal and dichotomous measures) and comparisons between randomised groups were analysed separately at 4 and 6 years using generalised linear models. Models were adjusted for minimisation covariates (age; type of prolapse repair planned; need for concomitant urinary incontinence procedure or not; need for concomitant upper vaginal prolapse procedure or not; and operating surgeon), baseline measures where appropriate and randomisation stratum. Continuous outcomes were analysed using linear mixed models with surgeon fitted as a random effect. Binary outcomes models used the binomial family and log link function while the ordinal outcomes were fitted with an ordinal regression model. The binary and ordinal models were adjusted for the minimisation covariates; however, in some cases due to small or nil category combination counts, some had to be dropped. Surgeon effect was adjusted for in the binary and ordinal outcome models using a cluster robust variance, which, while not the same as a random effects model (as for the continuous outcomes), has a similar effect.

PGI-I was analysed using ordinal logistic regression (proportional odds models with cumulative logits). Dichotomous outcomes were analysed using log-binomial regression. Estimates of treatment effect size were mean differences in the linear mixed models (including the analysis of the POP-SS), odds ratios in the ordinal models and risk ratios in the binary models. For all estimates, 95% confidence intervals were calculated.

### Definition of functional cure

Functional cure, at 6 years, was defined as a composite outcome, including all women who had not had a reintervention for prolapse during this follow-up period (surgery or pessary) and all women who answered “Never” to question 1 on the POP-SS (‘How often in the last 4 weeks have you had a feeling of something coming down from or in your vagina’) at 6 years.

## Results

### Participants and treatment

We randomised 1348 women from January 2010 to August 2013. Of the women randomised, 23 participants did not consent to be contacted for longer-term follow-up and 42 withdrew their consent from completing further questionnaires. These women were therefore excluded from the long-term follow-up study and did not receive the 4- and 6-year postal questionnaires.

**Fig. 1:**
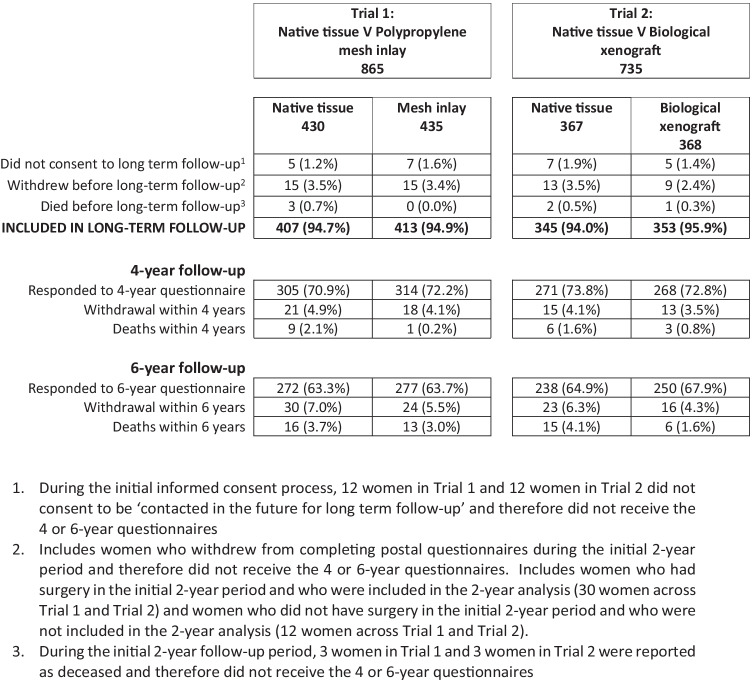
CONSORT diagram

Figure 1 (Consort Diagram the flow of women through the study at 4 and 6 years. The retention rates of those initially randomised remain high (72% at 4 years and 65% at 6 years), with no significant differential in drop-our rates. The baseline characteristics of responders and non-responders at 4 and 6 years are given in supplementary Table 1. Non-responders tended to be younger and have higher (worse) POP-SS scores at baseline, with lower (worse) EQ-5D-3L scores. However, other clinical characteristics were broadly similar and there was no difference in follow-up rates between randomised groups.

### Primary outcome

At 4 and 6 years, the mean POP-SS in the group randomised to a native tissue repair was lower (better) than those in the polypropylene mesh inlay or biological xenograft group (Table 1). The difference was only statistically significant for the polypropylene mesh inlay comparison to native tissue at 4 years, although the mean difference (MD) was small and less than the minimal clinically important difference (MCID) for the POP-SS at 4 and 6 years respectively (1.01 [95% CI 0.20–1.83], 0.85 [-0.05–1.75]).

**Table 1:** Prolapse urinary, bowel and dyspareunia symptoms at 1, 2, 4 and 6 years

	Trial 1: Native tissue repair vs polypropylene mesh inlay									Trial 2: Native tissue repair vs biological xenograft								
	Native tissue			Mesh inlay			Eff. Size	95% CI	p-value	Native tissue			Biological xenograft			Eff. size	95% CI	p-value
1-Year outcomes																		
No. of women at 1 year	N = 398			N = 391						N = 344			N = 340					
POP-SS at 1 year	5.4	(5.5)	395	5.5	(5.1)	389	0.04	-0.64 to 0.73	0.908	5.5	(5.6)	342	5.6	(5.6)	337	0.06	-0.71 to 0.83	0.876
Other measures of prolapse symptoms at 1 year																		
Symptomatic	83.0%	328	395	84.6%	329	389	1.02 [6]	0.95 to 1.09	0.563	82.7%	283	342	81.9%	276	337	0.99	0.93 to 1.05	0.643
Prolapse-related QoL score	2.0	(2.7)	389	2.2	(2.7)	380	0.13	-0.25 to 0.51	0.499	2.2	(2.8)	335	2.4	(2.9)	330	0.13	-0.30 to 0.56	0.544
2-Year outcomes																		
No. of women at 2 years	N = 348			N = 343						N = 299			N = 301					
POP-SS at 2 years	4.9	(5.1)	347	5.3	(5.1)	342	0.38	-0.31 to 1.08	0.278	4.9	(5.1)	298	5.6	(5.7)	300	0.57	-0.22 to 1.35	0.159
Symptomatic ^1^	81.6%	283	347	85.1%	291	342	1.04	0.98 to 1.11	0.194	81.2%	242	298	82.0%	246	300	1.01 [7]	0.94 to 1.08	0.756
Prolapse-related QoL score^2^	1.9	(2.5)	335	2.2	(2.6)	329	0.15	-0.23 to 0.54	0.435	2.0	(2.5)	290	2.1	(2.8)	292	0.09	-0.34 to 0.51	0.691
4-Year outcomes																		
No. of women at 4 years	N = 305			N = 314						N = 271			N = 268					
POP-SS at 4 years	5.1	(5.0)	297	6.1	(6.0)	311	1.01	0.20 to 1.83	0.015	5.3	(5.2)	266	5.8	(5.5)	262	0.54	-0.31 to 1.39	0.212
Other measures of prolapse symptoms at 4 years																		
Symptomatic ^1^	84.8%	252	297	85.2%	265	311	0.99	0.93 to 1.05	0.726	84.2%	224	266	85.5%	224	262	0.98	0.91 to 1.06	0.580
Prolapse-related QoL score^2^	2.1	(2.7)	290	2.4	(2.9)	310	0.35	-0.09 to 0.79	0.121	2.1	(2.6)	260	2.4	(2.8)	258	0.13	-0.34 to 0.59	0.593
Women with any report of SCD ^3^	35.4%	105	297	37.9%	118	311	1.02	0.85 to 1.23	0.819	35.7%	95	266	41.6%	109	262	1.15	0.97 to 1.35	0.100
Severe incontinence^4^	5.7%	17	296	5.3%	16	303	0.83	0.43 to 1.60	0.583	5.5%	14	256	8.3%	21	253	1.37	0.81 to 2.31	0.247
Severe faecal incontinence^5^	7.5%	22	292	9.0%	27	299	1.47 [8]	0.99 to 2.19	0.057	6.7%	17	253	7.7%	19	248	1.00 [9]	0.65 to 1.54	0.994
Dyspareunia^6^	5.6%	7	126	4.2%	5	119	0.80	0.21 to 2.95	0.731	5.0%	5	100	4.3%	5	115	0.81	0.22 to 3.00	0.756
6-Year outcomes																		
No. of women at 6 years	N = 272			N = 277						N = 238			N = 250					
POP_SS at 6 years	5.4	(5.4)	269	6.3	(6.2)	272	0.85	-0.05 to 1.75	0.063	5.9	(5.4)	237	6.2	(5.8)	248	0.27	-0.64 to 1.18	0.565
Other measures of prolapse symptoms at 6 years																		
Symptomatic ^1^	83.3%	224	269	85.3%	232	272	1.02	0.95 to 1.09	0.671	85.7%	203	237	85.9%	213	248	0.98	0.92 to 1.05	0.642
Prolapse-related QoL score^2^	2.3	(2.7)	264	2.5	(3.0)	264	0.26	-0.22 to 0.74	0.297	2.4	(2.8)	233	2.6	(3.0)	243	0.14	-0.38 to 0.65	0.605
Women with any report of SCD ^3^	37.2%	100	269	40.8%	111	272	1.13	0.95 to 1.33	0.165	40.5%	96	237	42.7%	106	248	1.13	0.92 to 1.40	0.251
Severe urinary incontinence^4^	6.8%	18	264	10.9%	29	267	1.58	0.97 to 2.59	0.067	6.6%	15	226	9.2%	22	238	1.36	0.73 to 2.52	0.333
Severe faecal incontinence^5^	9.3%	24	259	6.8%	18	266	0.73	0.45 to 1.17	0.186	9.5%	21	222	8.9%	21	237	0.86	0.53 to 1.38	0.523
Dyspareunia^6^	4.3%	4	93	5.7%	6	105	1.88	0.69 to 5.10	0.217	3.9%	3	76	6.9%	7	102	0.91	0.18 to 4.57	0.910

Footnotes:

1. Symptomatic = POP-SS > 0

2. Prolapse-related QoL score = quality of life due to prolapse symptoms measured as ‘overall interference of prolapse symptoms with everyday life’ using a visual analogue scale (VAS); score range from 0 (not at all) to 10 (a great deal)

3. Women with any report of SCD = ‘A feeling of something coming down from or in your vagina?’ (any = occasionally or more)

4. Severe urinary incontinence = International Consultation on Incontinence Questionnaire-Urinary Incontinence Short Form score of 13–21

5. Severe faecal incontinence = Faecal incontinence of solid or liquid stool: ‘Do stools (faeces, motion) leak at inappropriate time or place, or before you can get to the toilet?’ (severe = sometimes, most or all of the time)

6. Concomitant upper prolapse procedure and symptoms at baseline excluded due to nil group counts

7. Type of prolapse planned, concomitant upper prolapse procedure and symptoms at baseline excluded due to nil group counts

8. Concomitant upper prolapse procedure excluded because of nil group counts

9. Concomitant incontinence and concomitant upper prolapse procedure excluded because of nil group counts

There were no statistically significant differences in the number of women with at least one symptom of prolapse (defined as POP-SS > 0) or in quality-of-life related to prolapse symptoms.

### Other outcomes

#### Primary symptom of prolapse (something coming down [SCD])

There was no difference between groups in the number of women experiencing any degree of “something coming down” (Table 1). However, at 6 years, there appeared to be more women in the polypropylene mesh inlay group experiencing symptoms of SCD “frequently” compared to the native tissue group. This was not found in the biological xenograft group (Supplementary table 2).

#### Other pelvic floor symptoms

No significant statistical difference was found in the frequency of severe incontinence or faecal incontinence after surgery. All these symptoms improved compared to baseline rates. Dyspareunia rates fell across all groups (Table 1).

#### Functional cure (Table 2)

Using a composite outcome assessing functional cure at 6 years we found no difference in cure among the three types of surgery. Half the women were cured at 6 years but only 10.3 to 12% of women had undergone further surgery for prolapse. Some women will have had more than one further operation for prolapse by 6 years.

**Table 2:** 'Cured' along with any further treatment required by 6 years

	Trial 1: Native tissue repair vs polypropylene mesh inlay									Trial 2: Native tissue repair vs biological xenograft								
	Native tissue			Mesh inlay			Eff. size	95% CI	p-value	Native tissue			Biological xenograft			Eff. size	95% CI	p-value
6-Year outcomes																		
No. of women at 6 years	N = 272			N = 277						N = 238			N = 250					
Cured-no feeling of something coming down at 6 years, no prolapse surgery by 6 years, no pessary use at 6 years	53.7%	144	268	54.7%	150	274	1.02 [1]	0.88 to 1.19	0.797	51.5%	120	233	49.4%	122	247	0.99	0.83 to 1.18	0.892
Number of women having any new prolapse operation within 6 years (cumulative)	12.0%	32	266	10.3%	28	271	0.87	0.54 to 1.40	0.572	11.7%	27	231	11.8%	29	245	1.00	0.61 to 1.63	0.995
Number of women having any mesh removed within 6 years (cumulative)	1.6%	4	255	8.4%	22	263	5.24	1.84 to 14.96	0.002	1.4%	3	221	0.9%	2	231	0.59	0.10 to 3.48	0.562

Footnotes:

1. Concomitant incontinence procedure and concomitant upper prolapse procedure excluded because of nil group counts

#### Complications and adverse events

Over the 6-year period, further surgery to treat complications of the polypropylene mesh inlay was required by 8.4% of women in the mesh group, 0.9% of those in the native tissue group and 1.6 % of those in the biological xenograft group.

There was little difference between the arms in overall quality of life assessed using the EQ-5D-3L (Table 3). There was no significant difference between groups, in the number of women reporting generalised extreme pain or discomfort, which was assessed as part of EQ-5D- 3L.

**Table 3:** EQ-5D-3L and pain at 4 and 6 years

	Trial 1: Native tissue repair vs Polypropylene mesh inlay									Trial 2: Native tissue repair vs biological xenograft								
	Native tissue			Mesh inlay			Eff. size	95% CI	p-value	Native tissue			Biological xenograft			Eff. size	95% CI	p-value
4-Year outcomes																		
No. of women at 4 years	N = 305			N = 314						N = 271			N = 268					
EQ5D 3L	0.80	(0.27)	298	0.82	(0.23)	297	0.02	-0.02 to 0.05	0.288	0.80	(0.27)	265	0.82	(0.26)	262	0.02	-0.01 to 0.06	0.221
Pain/discomfort (extreme)	7.3	22	300	4.5	14	311	0.76 [1]	0.47 to 1.21	0.244	7.5	20	267	5.3	14	0	0.57	0.30 to 1.07	0.081
6-Year outcomes																		
No. of women at 6 years	N = 272			N = 277						N = 238			N = 250					
EQ5D 3L	0.80	(0.27)	263	0.81	(0.23)	271	-0.00	-0.04 to 0.04	0.903	0.80	(0.27)	229	0.80	(0.25)	244	0.01	-0.03 to 0.05	0.548
Pain/discomfort (extreme)	6.3	17	270	4.0	11	275	0.60 [1]	0.25 to 1.45	0.255	5.9	14	236	6.0	15	0	0.61 [2]	0.38 to 0.97	0.038

Footnotes:

1. Concomitant incontinence procedure and concomitant upper prolapse procedure excluded due to nil group counts

2. Age band, type of prolapse planned, concomitant incontinence procedure and concomitant upper prolapse procedure excluded because of nil group counts

#### Patient’s global impression of improvement and satisfaction (Table 4) and further treatment (Table 5)

Native tissue vs polypropylene mesh inlay

**Table 4:** Satisfaction with surgery at 4 and 6 years

	Trial 1: Native tissue repair vs polypropylene mesh inlay								Trial 2: Native tissue repair vs biological xenograft									
	Native tissue			Mesh inlay		Eff. size		95% CI	p- value	Native tissue		Biological xenograft				Eff. size	95% CI	p- value
4-Year outcomes																		
No. of women at 4 years	N = 305			N = 314						N = 271			N = 268					
Prolapse compared with before surgery at 4 years																		
Very much better	50.4%	143	284	47.4%	135	285	1.22	0.94 to 1.58	0.129	51.6%	132	256	44.4%	111	250	1.46	1.14 to 1.87	0.003
Much better	28.2%	80	284	26.7%	76	285				25.0%	64	256	20.8%	52	250			
A little better	10.2%	29	284	9.8%	28	285				11.3%	29	256	14.8%	37	250			
No change	4.2%	12	284	6.0%	17	285				4.3%	11	256	12.0%	30	250			
A little worse	2.8%	8	284	3.5%	10	285				3.9%	10	256	6.8%	17	250			
Much worse	2.8%	8	284	4.2%	12	285				3.1%	8	256	0.8%	2	250			
Very much worse	1.4%	4	284	2.5%	7	285				0.8%	2	256	0.4%	1	250			
Satisfaction with surgery at 4 years																		
Completely satisfied	58.2%	167	287	51.5%	151	293	1.37	1.04 to 1.80	0.023	59.1%	152	257	47.5%	121	255	1.58	1.16 to 2.15	0.004
Fairly satisfied	28.9%	83	287	30.7%	90	293				28.0%	72	257	32.5%	83	255			
Fairly dissatisfied	4.2%	12	287	5.5%	16	293				4.7%	12	257	8.6%	22	255			
Very dissatisfied	5.9%	17	287	9.2%	27	293				5.1%	13	257	6.3%	16	255			
Not sure	2.8%	8	287	3.1%	9	293				3.1%	8	257	5.1%	13	255			
Recommend to a friend at 4 years																		
Yes	90.3%	250	277	87.9%	239	272	0.98[1]	0.92 to 1.03	0.430	91.1%	225	247	87.6%	212	242	0.95 [2]	0.91 to 0.98	0.003
6-Year outcomes																		
No. of women at 6 years	N = 272			N = 277						N = 238			N = 250					
Prolapse compared with before surgery at 6 years																		
Very much better	46.7%	119	255	41.9%	111	265	1.41	1.07 to 1.86	0.016	45.2%	100	221	37.6%	88	234	1.36	0.93 to 1.98	0.113
Much better	30.6%	78	255	27.2%	72	265				29.4%	65	221	29.9%	70	234			
A little better	9.0%	23	255	8.7%	23	265				10.4%	23	221	12.8%	30	234			
No change	6.3%	16	255	8.7%	23	265				6.8%	15	221	8.1%	19	234			
A little worse	4.3%	11	255	7.2%	19	265				4.5%	10	221	6.4%	15	234			
Much worse	2.0%	5	255	2.6%	7	265				1.8%	4	221	3.8%	9	234			
Very much worse	1.2%	3	255	3.8%	10	265				1.8%	4	221	1.3%	3	234			
Satisfaction with surgery at 6 years																		
Completely satisfied	55.4%	144	260	42.9%	115	268	1.67	1.31 to 2.13	0.000	54.8%	125	228	46.2%	108	234	1.26	0.85 to 1.87	0.255
Fairly satisfied	30.4%	79	260	35.4%	95	268				29.4%	67	228	35.0%	82	234			
Fairly dissatisfied	3.1%	8	260	8.6%	23	268				3.1%	7	228	8.5%	20	234			
Very dissatisfied	5.8%	15	260	8.6%	23	268				5.3%	12	228	6.4%	15	234			
Not sure	5.4%	14	260	4.5%	12	268				7.5%	17	228	3.8%	9	234			
Recommend to a friend at 6 years																		
Yes	89.0%	219	246	84.8%	217	256	0.93[3]	0.90 to 0.98	0.002	89.7%	192	214	85.2%	184	216	0.95 [2]	0.90 to 1.01	0.084

Footnotes:

1. Concomitant upper prolapse procedure excluded because of nil group counts

2. Type of prolapse planned, concomitant incontinence and concomitant upper prolapse procedure excluded because of nil group counts

3. Concomitant incontinence and concomitant upper prolapse procedure excluded because of nil group counts

**Table 5:** Further treatment required at 4 and 6 years

	Trial 1: Native tissue repair vs polypropylene mesh inlay									Trial 2: Native tissue repair vs biological xenograft								
	Native tissue			Mesh inlay			Eff. size	95% CI	p-value	Native tissue			Biological xenograft			Eff. size	95% CI	p-value
4-Year outcomes																		
No. of women at 4 years	N=305			N=314						N=271			N=268					
New prolapse operation ^1^	4.0%	12	299	2.9%	9	311	0.76	0.36 to 1.61	0.471	3.0%	8	264	4.5%	12	266	1.44	0.77 to 2.67	0.253
Continence surgery	2.0%	6	300	0.7%	2	307	0.35	0.07 to 1.75	0.202	1.1%	3	268	0.4%	1	267	0.31	0.03 to 3.07	0.319
-SUI ^2^	1.3%	4	300	0.7%	2	307				0.4%	1	268	.%	.	267			
-UI ^3^	0.7%	2	300	.%	.	307				0.7%	2	268	0.4%	1	267			
Mesh removed ^4^	1.1%	3	285	3.9%	12	305	3.42	1.01 to 11.60	0.049	0.8%	2	259	0.4%	1	257	0.55	0.05 to 6.29	0.630
Readmitted to hospital	1.7%	5	302	2.3%	7	309	1.43	0.49 to 4.16	0.513	2.2%	6	269	.%	.	261			
-POP other ^5^	1.0%	3	302	1.6%	5	309				1.5%	4	269	.%	.	261			
-UI other ^6^	0.7%	2	302	0.6%	2	309				0.7%	2	269	.%	.	261			
6-Year outcomes																		
No. of women at 6 years	N = 272			N = 277						N = 238			N = 250					
New prolapse operation ^1^	2.6%	7	272	2.5%	7	275	1.02	0.36 to 2.87	0.972	2.5%	6	238	3.6%	9	248	1.47	0.84 to 2.57	0.174
Continence surgery	1.9%	5	270	0.4%	1	276	0.24	0.02 to 2.43	0.227	2.1%	5	236	0.4%	1	249	0.18	0.02 to 1.80	0.144
-SUI ^2^	0.7%	2	270	.%	.	276				0.8%	2	236	0.4%	1	249			
-UI ^3^	1.1%	3	270	0.4%	1	276				1.3%	3	236	.%	.	249			
Mesh removed ^4^	0.7%	2	268	2.2%	6	269	2.95	0.69 to 12.70	0.146	0.4%	1	232	0.4%	1	240	0.89	0.04 to 22.16	0.945
Readmitted to hospital	0.4%	1	268	1.1%	3	277	2.99	0.20 to 44.51	0.427	0.4%	1	236	0.4%	1	247	0.85	0.08 to 8.56	0.888
-POP other ^5^	0.4%	1	268	0.7%	2	277				0.4%	1	236	.%	.	247			
-UI other ^6^	0%	0	268	0.4%	1	277				0%	0	236	0.4%	1	247			

Footnotes:

1. Repeat prolapse surgery: anterior or posterior repair, vaginal hysterectomy (if for prolapse), vault repair, sacrospinous fixation

2. Surgery for SUI: tape, midurethral sling, colposuspension, traditional sling, anterior repair (if for SUI), other

3. Other surgery for UI (any UI/urgency UI): Botox, sacral nerve stimulation (SNS), injectables, other

4. Any admission for mesh removal or mesh problems

5. Any other admission for prolapse-related problems: pessaries, granulation tissue procedures, Fenton’s procedure (for tight vagina), adhesion removal, other

6. Any other admission for urinary tract-related problems: UTIs, urinary retention, cystoscopy (if thought to be related to previous POP or continence surgery), catheter problems, other

At 4 years there was no difference found in women’s perceptions of improvement in prolapse symptoms. However, those who had undergone a native tissue repair were significantly more satisfied with the overall outcome of their surgery. By 6 years, significantly more of the native tissue group felt their symptoms were very much better and they were overall more satisfied with the outcome of their operation than the polypropylene mesh inlay group.

Native tissue vs biological xenograft

At 4 years following surgery the native tissue group were both more satisfied and perceived the prolapse symptoms were better than the biological xenograft graft group. However, by 6 years there was no difference in prolapse symptoms or overall satisfaction.

Table 5 demonstrates there were no significant difference in further treatment rates for prolapse or SUI.

## Dicussion

### Main findings

At 6 years following surgery for vaginal wall prolapse, our study found no clinical or statistically significant difference in the trial's primary outcome, POP-SS score.

The overall cure rate at 6 years, a composite of no symptoms of SCD, no use of pessaries and no further surgery for prolapse, was approximately 50% for all methods.

We found no difference in rates of “generic pain” or dyspareunia, both of which were low. In addition, the was no difference in overall quality of life between groups

We also found no difference in any of the outcomes commonly used to assess prolapse surgery among the three methods of prolapse repair (native tissue, polypropylene mesh inlay or a biological xenograft) except for PGI-I, global satisfaction and SCD ‘frequently’, although not consistently over the different time points (Table 4).

### Meaning of the study

It appears neither polypropylene mesh inlays nor biological xenografts offer any additional benefit to the outcome of prolapse surgery at 4–6 years.

At this mid-term point, women having polypropylene mesh inlays possibly appear to have slightly worse outcomes compared to those having native tissue repairs. They appear to have a higher incidence of “SCD frequently” and higher overall dissatisfaction. Their general dissatisfaction could be influenced by the negative publicity surrounding polypropylene mesh or the 8.4% who required mesh excision surgery. Other studies in which women have been examined, although small and underpowered, found a higher incidence of prolapse in the non-operated compartment in the polypropylene mesh group compared to the native tissue group [1, 2]. Another important factor of note is that the slightly increased general dissatisfaction in the polypropylene mesh inlay group does not appear to be due to any difference in the incidence of pain, dyspareunia or worse quality of life between polypropylene mesh inlays and native tissue repairs.

Pelvic organ prolapse is a very complex problem to assess because of variations in: the incidence and treatment in the three compartments of the vagina, the high long-term recurrence rate, the undefined variation in individual surgical techniques, lack of consistency in the examination techniques, especially the force exerted on the pelvic floor during examination, and the large range of pelvic floor symptoms, which may or may not be caused by the prolapse. Hence at present, large multicentre RCTs addressing global treatment and using validated patient-reported outcomes (PROMs) are probably the most useful trial designs to compare treatments. These studies should produce the most robust, generalisable and least biased information, although their limitation is that they fail to explain the pathophysiology of recurrent prolapse.

Controversy remains about the best outcome measure for pelvic organ prolapse surgery and how to define cure. Success varies depending on the outcome chosen. PGI-I is useful to capture a global overview but does not help to identify the reasons for failure. It is simple to administer and used commonly by many studies. Is the selection of “much better” on PGI-I equivalent to cure ? Or is “SCD never” a more appropriate definition of cure? Repeat operation rates should probably not be used to define success as there are many factors which may prevent women from seeking further surgery. Satisfaction may be influenced by many factors including pre-operative counselling. Again, this highlights the strength of our large RCT in directly comparing these outcomes.

Trials need to be large enough to account for the significant long-term follow-up issues which will occur not only from trial fatigue but also from the increasing frailty and mortality of an ageing population.

To date only four RCTs assessing vaginal surgery for prolapse with mid-term (5-7 years) follow-up have been published [1, 2, 15, 16]. Three compared polypropylene mesh kits to native tissue repairs and one compared allograft to native tissue repairs. All the studies were small, with < 200 participants. They were all powered to assess large differences in anatomical outcomes. However, in their long-term follow-up phase, most changed their primary outcome and chose to report PROMs instead. Hence, they all have a significant risk of bias because they are underpowered.

Only one study is directly comparable to PROSPECT, performed in women having primary surgery in 12 centres across France [2, 17]. At 1 year they concluded mesh vaginal wall prolapse repairs give better 1-year anatomical results than traditional colporrhaphy. However, there is no difference in the later follow-up phase. Allegre’s trial in primary cases using mesh kits found there was no benefit from mesh kits over native tissue repairs at 5 to 8 years follow-up [2].

Our composite success rate was comparable to the other trials even though they included anatomical data. Milani et al. [1] used a composite outcome of (1) presence of no bulge on POP-Q [18], (2) no symptom of bulge and (3) no further treatment. They found native tissue cure rate was 54% and polypropylene mesh was 53% at 7 years.

Milani et al. [1] found anatomical recurrence in the non-mesh compartment for those who had transvaginal mesh and in the operative compartment for those who had native tissue repair. Allegre et al. [2] also found an anatomical failure rate of 67% in the native tissue group compared to only 24% in polypropylene mesh group.

The interpretation of polypropylene mesh complications in PROSPECT is limited by the lack of physical examination. There will be some women who have undiagnosed asymptomatic exposures. In PROSPECT, 8.4% of women in the polypropylene mesh group required further surgery for mesh exposure. This is very similar to other studies; Allegre et al. [2] found 8%, Milani et al. [1] 13% and Heinonen et al. [19] in a large case series found 11.2% required further surgery.

In PROSPECT, the background rate of polypropylene mesh complications in the native tissue and biological xenograft groups, arising from polypropylene mesh inserted at another time, was 0.9% and 1.6% respectively. This background rate may not be accounted for in case series.

One of PROSPECT's strengths was the ability of the RCT design to compare complications to native tissue repairs. Of note there was also no difference in dyspareunia or general pain (assessed by EQ5D) between groups. Milani et al. and Allegre et al. also found no significant difference in pain or dyspareunia between the groups [1, 2]. Our recent publication assessing the whole cohort of women in PROSPECT (primary, secondary and cohort study) at 2 years also found no difference [7]. This raises the possibility that total polypropylene mesh removal surgery for these indications may not be beneficial and this requires urgent further research.

### Strengths and weaknesses

The strength of PROSPECT remains its size, generalisability and robust methodology. Unlike other studies criticised in the Cumberlege report [8] for being sponsored by industry, PROSPECT was independent, funded by the UK National Institute for Health Research (NIHR) Health Technology Assessment (HTA) programme.

Due to the average age of women who undergo prolapse surgery, all prolapse studies will encounter issues with long-term follow-up due to unrelated mortality, increasing co-morbidity and frailty, which should occur equally across all randomised groups. This is one of the reasons RCTs provide more accurate results than case series, registries or cohort studies. At 6 years we have maintained follow-up for the primary outcome of the trial in 65% of women.

In addition, the proportion of women having concomitant upper compartment prolapse or continence surgery were evenly distributed between the randomised groups and therefore did not have an impact on the findings.

Notably, the pain and dyspareunia rates were similar across all groups. This highlights the importance of RCT methods to evaluate conditions. Case series may provide a biased impression of complication rates if there is no comparator group [20, 21]. However, RCTs can never be large enough to detect rare complications which require large population-based epidemiological studies. Such studies do then need to be followed by biological studies to determine causation versus association.

One of PROSPECT’s weaknesses was the lack of anatomical data, although one could argue that anatomical data matters little to women for whom symptoms are paramount. Currently, there is also inconsistency in evaluation of anatomical outcomes due to lack of standardisation of strain or time of measurement, which will reduce their validity. However, anatomical data may help us to understand the mechanism of failure in the different procedures. Anatomical data were not collected at 4 and 6 years for two reasons. The first was the significant increased financial cost of physical assessments. The second was concern about the risk of trial fatigue. Hence, physical examination was planned to be limited to the end point at 12 years. This time frame was chosen using the very limited data available on long-term recurrence of prolapse (Olsen) [3].

The study included many different types of polypropylene mesh and biological xenograft, which some clinicians feel is a weakness because they believe in the performance of certain products in their hands. However, there will always be companies who reproduce other similar devices used by surgeons with a variety of training and experience. Hence, the real-world pragmatic approach of PROSPECT is much more useful to women and health service planners.

PROSPECT was initially criticised for choosing to use polypropylene mesh inlays as opposed to mesh kits. However, most mesh kits have since been withdrawn from the market and therefore knowledge about the efficacy of inlays has become more important. Also, following the recommendations of NICE [22] and the Cumberlege report [8], some surgeons may consider using biological xenografts instead of polypropylene mesh inlays. However, our trial suggests there is no benefit of these over native tissue repairs.

### Future research

We consider our trial to have only reached mid-term follow-up because the seminal paper by Olsen et al., published 24 years ago, found the mean time between the first and second operation was 12 years [3]. Hence, there is a possibility that potential benefits of polypropylene mesh may not be seen until this time point. Longevity of a procedure and the effects of repeat surgery are important, especially considering the prediction that the mean life expectancy of the population will continue to increase.

The overall composite failure rate in most studies remains high, and although the repeat surgery rate is lower, a need remains for greater understanding of why prolapse surgery fails. Is it due to failure of surgeons to address apical support, variations in lifestyle, connective tissue types, intra-abdominal pressure, neurological factors, failure of the perineal body or anococcygeal raphe leading to a change in the angle of the pelvic floor?

Concerns remain among some women and clinicians that polypropylene mesh may cause chronic pain or be associated with fibromyalgia [8]. It was therefore reassuring that we found no significant difference in the generic quality of life of women, measured using the EQ5D, including the domain which assesses general pain. However, if a complication was very rare, our study would not be large enough to capture such an event.

### Conclusions

At 6 years there is no benefit from augmenting primary prolapse repairs with polypropylene mesh inlays or biological xenografts. There was no evidence that polypropylene mesh inlays caused greater pain or dyspareunia than native tissue repairs.

A need remains for long-term follow-up and further research into the pathophysiology of prolapse.

## Supplementary Information

ESM 1 (DOCX 44 kb)
