# Supplementary material for: PROSPECT: 4- and 6-year follow-up of a randomised trial of surgery for vaginal prolapse
Source: Int Urogynecol J. 2022 Aug 26;34(1):67–78. doi: 10.1007/s00192-022-05308-0 (PMC9834125; doi:10.1007/s00192-022-05308-0)
Supplement: Supplementary file 1 — (DOCX 44 kb) [file 192_2022_5308_MOESM1_ESM.docx]

**Supplementary Table 1: Baseline characteristics of responders and non-responders at 4 and 6 years**

| **Baseline Characteristics** |  | | | | | | | | | | | |
| --- | --- | --- | --- | --- | --- | --- | --- | --- | --- | --- | --- | --- |
|  | **4 year** | | | | | | **6 year** | | | | | |
|  | **Responders**  **(979)** | | | **Non-responders**  **(264)** | | | **Responders**  **(878)** | | | **Non-responders**  **(320)** | | |
| **Age** | 60.5 | (9.6) | 979 | 54.8 | (11.2) | 264 | 60.7 | (9.3) | 878 | 54.6 | (11.0) | 320 |
| **POP-SS** | 13.2 | (5.7) | 935 | 15.4 | (6.1) | 246 | 13.2 | (5.7) | 840 | 14.8 | (6.1) | 298 |
| **EQ-5D-3L** score | 0.73 | (0.22) | 911 | 0.67 | (0.28) | 236 | 0.74 | (0.21) | 821 | 0.68 | (0.27) | 286 |
| **Pain/discomfort (extreme) from EQ5D** | 1.83 | (0.51) | 925 | 1.95 | (0.56) | 241 | 1.83 | (0.50) | 831 | 1.95 | (0.55) | 294 |
| **Types of PLANNED Prolapse Surgery** |  |  |  |  |  |  |  |  |  |  |  |  |
| Anterior only | 45.4% | 444 | 979 | 43.2% | 114 | 264 | 45.9% | 403 | 878 | 43.4% | 139 | 320 |
| Posterior only | 26.0% | 255 | 979 | 27.3% | 72 | 264 | 26.7% | 234 | 878 | 24.4% | 78 | 320 |
| Both anterior and posterior | 28.6% | 280 | 979 | 29.5% | 78 | 264 | 27.4% | 241 | 878 | 32.2% | 103 | 320 |
| **PLANNED Concomitant Prolapse Surgery** |  |  |  |  |  |  |  |  |  |  |  |  |
| Vaginal hysterectomy | 35.6% | 349 | 979 | 32.6% | 86 | 264 | 36.0% | 316 | 878 | 32.2% | 103 | 320 |
| Abdominal hysterectomy | 0.4% | 4 | 979 | .% | . | 264 | 0.3% | 3 | 878 | 0.3% | 1 | 320 |
| Cervical amputation | 1.5% | 15 | 979 | 2.3% | 6 | 264 | 1.6% | 14 | 878 | 2.2% | 7 | 320 |
| Vault repair | 14.9% | 146 | 979 | 17.0% | 45 | 264 | 15.0% | 132 | 878 | 16.3% | 52 | 320 |
| **Concomitant continence operation** | 11.6% | 114 | 979 | 9.1% | 24 | 264 | 10.7% | 94 | 878 | 12.5% | 40 | 320 |
| % n/N for categorical, mean (SD) N for continuous variable | | | | | | | | | | | | |

**Supplementary Table 2: Individual prolapse symptoms at four and six years**

|  | **Trial 1: Native tissue repair v Polypropylene mesh inlay** | | | | | | | | | **Trial 2: Native tissue repair v Biological xenograft** | | | | | | | | |
| --- | --- | --- | --- | --- | --- | --- | --- | --- | --- | --- | --- | --- | --- | --- | --- | --- | --- | --- |
| **Variable** | **Native tissue** | | | **Mesh inlay** | | | **Eff. Size** | **95% CI** | **p-value** | **Native tissue** | | | **Biological xenograft** | | | **Eff. Size** | **95% CI** | **p-value** |
| **4-year outcomes** | | | | | | | | | | | | | | | | | | |
| **Number of Women at 4 years** | **N=305** | | | **N=314** | | |  |  |  | **N=271** | | | **N=268** | | |  |  |  |
| **Individual prolapse symptoms** | | | | | | | | | | | | | | | | | | |
| Women with any report of SCD^1^ | 35.4% | 105 | 297 | 37.9% | 118 | 311 | 1.02 | 0.85 to 1.23 | 0.819 | 35.7% | 95 | 266 | 41.6% | 109 | 262 | 1.15 | 0.97 to 1.35 | 0.100 |
| SCD -freq^2^ | 7.1% | 21 | 297 | 11.9% | 37 | 311 | 1.60 | 1.01 to 2.53 | 0.046 | 6.8% | 18 | 266 | 11.5% | 30 | 262 | 1.20 | 0.73 to 1.99 | 0.476 |
| Vaginal pain (standing) -any | 22.9% | 68 | 297 | 28.9% | 90 | 311 |  |  |  | 23.7% | 63 | 266 | 29.4% | 77 | 262 |  |  |  |
| Heavy/dragging in abdomen- any | 30.6% | 91 | 297 | 38.6% | 120 | 311 |  |  |  | 31.2% | 83 | 266 | 39.3% | 103 | 262 |  |  |  |
| Heavy/dragging in back -any | 36.7% | 109 | 297 | 43.1% | 134 | 311 |  |  |  | 37.2% | 99 | 266 | 38.5% | 101 | 262 |  |  |  |
| Strain to empty bladder - any | 43.8% | 130 | 297 | 45.0% | 140 | 311 |  |  |  | 39.1% | 104 | 266 | 47.3% | 124 | 262 |  |  |  |
| Bladder does not empty - any | 58.9% | 175 | 297 | 63.0% | 196 | 311 |  |  |  | 58.6% | 156 | 266 | 63.0% | 165 | 262 |  |  |  |
| Bowel does not empty - any | 68.0% | 202 | 297 | 70.1% | 218 | 311 |  |  |  | 67.7% | 180 | 266 | 66.0% | 173 | 262 |  |  |  |
| **Actions necessities by prolapse symptoms** | | | | | | | | | | | | | | | | | | |
| Finger to ease discomfort | 1.4% | 4 | 293 | 1.9% | 6 | 309 |  |  |  | 1.1% | 3 | 262 | 0.4% | 1 | 259 |  |  |  |
| Extra hygiene measure | 5.4% | 16 | 295 | 5.9% | 18 | 307 |  |  |  | 5.3% | 14 | 265 | 6.2% | 16 | 260 |  |  |  |
| Finger to empty bladder | 0.3% | 1 | 300 | 0.6% | 2 | 312 |  |  |  | 0.4% | 1 | 266 | 1.1% | 3 | 265 |  |  |  |
| Finger to empty bowel | 2.7% | 8 | 294 | 2.6% | 8 | 309 |  |  |  | 3.4% | 9 | 261 | 1.9% | 5 | 264 |  |  |  |
| Digital evacuation of bowel | 2.7% | 8 | 299 | 2.6% | 8 | 308 |  |  |  | 3.4% | 9 | 265 | 1.9% | 5 | 266 |  |  |  |
|  |  |  |  |  |  |  |  |  |  |  |  |  |  |  |  |  |  |  |
| **6-year outcomes** | | | | | | | | | | | | | | | | | | |
| **Number of Women at 6 years** | **N=272** | | | **N=277** | | |  |  |  | **N=238** | | | **N=250** | | |  |  |  |
| **Individual prolapse symptoms** | | | | | | | | | | | | | | | | | | |
| Women with any report of SCD^1^ | 37.2% | 100 | 269 | 40.8% | 111 | 272 | 1.13 | 0.95 to 1.33 | 0.165 | 40.5% | 96 | 237 | 42.7% | 106 | 248 | 1.13 | 0.92 to 1.40 | 0.251 |
| SCD -freq | 6.7% | 18 | 269 | 13.6% | 37 | 272 | 2.09 | 1.48 to 2.96 | 0.000 | 7.6% | 18 | 237 | 9.3% | 23 | 248 | 1.04 | 0.49 to 2.19 | 0.924 |
| Vaginal pain (standing) -any | 21.6% | 58 | 269 | 34.2% | 93 | 272 |  |  |  | 26.2% | 62 | 237 | 27.4% | 68 | 248 |  |  |  |
| Heavy/dragging in abdomen- any | 32.7% | 88 | 269 | 36.0% | 98 | 272 |  |  |  | 34.6% | 82 | 237 | 37.1% | 92 | 248 |  |  |  |
| Heavy/dragging in back -any | 37.5% | 101 | 269 | 40.8% | 111 | 272 |  |  |  | 40.5% | 96 | 237 | 38.3% | 95 | 248 |  |  |  |
| Strain to empty bladder - any | 45.7% | 123 | 269 | 47.4% | 129 | 272 |  |  |  | 45.6% | 108 | 237 | 48.0% | 119 | 248 |  |  |  |
| Bladder does not empty - any | 61.0% | 164 | 269 | 66.9% | 182 | 272 |  |  |  | 64.1% | 152 | 237 | 65.3% | 162 | 248 |  |  |  |
| Bowel does not empty - any | 67.3% | 181 | 269 | 69.9% | 190 | 272 |  |  |  | 71.7% | 170 | 237 | 67.7% | 168 | 248 |  |  |  |
| **Actions necessities by prolapse symptoms** | | | | | | | | | | | | | | | | | | |
| Finger to ease discomfort | 1.1% | 3 | 261 | 2.6% | 7 | 272 |  |  |  | 1.7% | 4 | 229 | 1.2% | 3 | 245 |  |  |  |
| Extra hygiene measure | 2.7% | 7 | 262 | 8.1% | 22 | 271 |  |  |  | 4.4% | 10 | 229 | 6.9% | 17 | 245 |  |  |  |
| Finger to empty bladder | 0.4% | 1 | 269 | 1.1% | 3 | 273 |  |  |  | 0.9% | 2 | 235 | 0.8% | 2 | 246 |  |  |  |
| Finger to empty bowel | 3.1% | 8 | 261 | 3.3% | 9 | 270 |  |  |  | 2.6% | 6 | 230 | 2.9% | 7 | 245 |  |  |  |
| Digital evacuation of bowel | 3.8% | 10 | 266 | 3.6% | 10 | 274 |  |  |  | 3.0% | 7 | 233 | 3.6% | 9 | 249 |  |  |  |

Footnotes:

1. Women with any report of SCD = ‘A feeling of something coming down from or in your vagina? (any = occasionally or more)
2. SCD freq = A feeling of something coming down from or in your vagina? (frequent = most or all of the time)

**Supplementary Table 3: Further conservative treatment required at 4 and 6 years**

|  | **Trial 1: Native tissue repair v Polypropylene mesh inlay** | | | | | | | | | **Trial 2: Native tissue repair v Biological**  **xenograft** | | | | | | | | |
| --- | --- | --- | --- | --- | --- | --- | --- | --- | --- | --- | --- | --- | --- | --- | --- | --- | --- | --- |
|  | Native tissue | | | Mesh inlay | | | Eff. size | 95% CI | p-value | Native tissue | | | Biological xenograft | | | Eff. size | 95% CI | p-value |
| **4-year outcomes** |  |  |  |  |  |  |  |  |  |  |  |  |  |  |  |  |  |  |
| **No. of women at 4 years** | **N=305** |  |  | **N=314** |  |  |  |  |  | **N=271** |  |  | **N=268** |  |  |  |  |  |
|  |  |  |  |  |  |  |  |  |  |  |  |  |  |  |  |  |  |  |
| *Treatment for Prolapse at 4 years* | | | | | | | | | | | | | | | | | | |
| Medicines for prolapse | 7.4% | 22 | 297 | 9.6% | 30 | 311 |  |  |  | 9.1% | 24 | 265 | 5.3% | 14 | 263 |  |  |  |
| -Oestrogen | 10.5% | 32 | 305 | 11.5% | 36 | 314 |  |  |  | 14.0% | 38 | 271 | 12.3% | 33 | 268 |  |  |  |
| -Ring Pessary | 2.0% | 6 | 305 | 3.2% | 10 | 314 |  |  |  | 2.6% | 7 | 271 | 2.2% | 6 | 268 |  |  |  |
| -Shelf Pessary | 1.6% | 5 | 305 | 1.6% | 5 | 314 |  |  |  | 2.6% | 7 | 271 | 0.4% | 1 | 268 |  |  |  |
| Physiotherapy | 1.7% | 5 | 295 | 1.6% | 5 | 312 |  |  |  | 1.9% | 5 | 264 | 2.3% | 6 | 263 |  |  |  |
| GP for prolapse | 10.7% | 31 | 291 | 13.7% | 42 | 307 |  |  |  | 10.0% | 26 | 261 | 7.6% | 20 | 263 |  |  |  |
| Practice Nurse for prolapse | 2.4% | 7 | 295 | 2.6% | 8 | 308 |  |  |  | 1.1% | 3 | 264 | 1.1% | 3 | 263 |  |  |  |
| Hospital Outpatients for prolapse | 10.8% | 32 | 295 | 9.4% | 29 | 310 |  |  |  | 11.0% | 29 | 264 | 5.0% | 13 | 261 |  |  |  |
|  |  |  |  |  |  |  |  |  |  |  |  |  |  |  |  |  |  |  |
| *Treatment for urinary problems at 4 years* | | | | | | | | | | | | | | | | | | |
| Absorbent Pads | 28.0% | 84 | 300 | 34.0% | 106 | 312 |  |  |  | 29.5% | 79 | 268 | 26.6% | 70 | 263 |  |  |  |
| Permanent Catheter | 0% | 0 | 295 | 0.3% | 1 | 299 |  |  |  |  |  |  |  |  |  |  |  |  |
| Intermittent Catheter | 1.7% | 5 | 302 | 1.9% | 6 | 313 |  |  |  | 1.9% | 5 | 269 | 1.5% | 4 | 266 |  |  |  |
| Drugs for UI | 7.2% | 22 | 305 | 8.3% | 26 | 314 |  |  |  | 7.7% | 21 | 271 | 4.1% | 11 | 268 |  |  |  |
|  |  |  |  |  |  |  |  |  |  |  |  |  |  |  |  |  |  |  |
| **6-year outcomes** |  |  |  |  |  |  |  |  |  |  |  |  |  |  |  |  |  |  |
| **No. of women at 6 years** | **N=272** |  |  | **N=277** |  |  |  |  |  | **N=238** |  |  | **N=250** |  |  |  |  |  |
|  |  |  |  |  |  |  |  |  |  |  |  |  |  |  |  |  |  |  |
| *Treatment for Prolapse at 6 years* | | | | | | | | | | | | | | | | | | |
| Medicines for prolapse | 7.8% | 21 | 269 | 8.0% | 22 | 275 |  |  |  | 6.8% | 16 | 237 | 4.5% | 11 | 247 |  |  |  |
| -Oestrogen | 11.4% | 31 | 272 | 13.0% | 36 | 277 |  |  |  | 11.3% | 27 | 238 | 13.2% | 33 | 250 |  |  |  |
| -Ring Pessary | 3.3% | 9 | 272 | 4.0% | 11 | 277 |  |  |  | 2.5% | 6 | 238 | 1.6% | 4 | 250 |  |  |  |
| -Shelf Pessary | 1.1% | 3 | 272 | 0.4% | 1 | 277 |  |  |  | 1.7% | 4 | 238 | 2.0% | 5 | 250 |  |  |  |
| Physiotherapy | 1.5% | 4 | 266 | 3.6% | 10 | 275 |  |  |  | 2.1% | 5 | 233 | 3.2% | 8 | 247 |  |  |  |
| GP for prolapse | 9.1% | 24 | 265 | 12.9% | 35 | 271 |  |  |  | 10.7% | 25 | 233 | 11.0% | 27 | 245 |  |  |  |
| Practice Nurse for prolapse | 4.5% | 12 | 265 | 3.7% | 10 | 271 |  |  |  | 4.7% | 11 | 232 | 2.9% | 7 | 243 |  |  |  |
| Hospital Outpatients for prolapse | 8.2% | 22 | 267 | 8.5% | 23 | 272 |  |  |  | 9.0% | 21 | 233 | 6.5% | 16 | 247 |  |  |  |
| *Treatment for urinary problems at 6 years* | | | | | | | | | | | | | | | | | | |
| Absorbent Pads | 34.2% | 92 | 269 | 41.1% | 113 | 275 |  |  |  | 34.9% | 82 | 235 | 35.2% | 88 | 250 |  |  |  |
| Permanent Catheter | 0% | 0 | 263 | 0.4% | 1 | 270 |  |  |  |  |  |  |  |  |  |  |  |  |
| Intermittent Catheter | 1.5% | 4 | 269 | 0.7% | 2 | 274 |  |  |  | 1.7% | 4 | 236 | 1.2% | 3 | 249 |  |  |  |
| Drugs for UI | 6.3% | 17 | 272 | 9.0% | 25 | 277 |  |  |  | 8.4% | 20 | 238 | 5.6% | 14 | 250 |  |  |  |
